# Deep dissection of the antiviral immune profile of patients with COVID-19

**DOI:** 10.1038/s42003-021-02852-1

**Published:** 2021-12-16

**Authors:** Djordje Atanackovic, Stephanie V. Avila, Forat Lutfi, Diego de Miguel-Perez, Xiaoxuan Fan, Gabriela Sanchez-Petitto, Erica Vander Mause, Jonathan Siglin, John Baddley, Heather D. Mannuel, Hanan Alkhaldi, Kim G. Hankey, Rena Lapidus, Michael Kleinberg, Joseph Rabin, Carl Shanholtz, Christian Rolfo, Aaron P. Rapoport, Saurabh Dahiya, Tim Luetkens

**Affiliations:** 1 Transplant and Cellular Therapy Program, Department of Medicine, University of Maryland School of Medicine and Greenebaum Comprehensive Cancer Center, Baltimore, MD USA; 2 Department of Microbiology and Immunology, University of Maryland, Baltimore, MD USA; 3 University of Maryland School of Medicine and Greenebaum Comprehensive Cancer Center, Baltimore, MD USA; 4 Division of Infectious Diseases, University of Maryland School of Medicine and Greenebaum Comprehensive Cancer Center, Baltimore, MD USA; 5 Hematology/Oncology, University of Maryland Marlene and Stewart Greenebaum Comprehensive Cancer Center, Baltimore, MD USA; 6 Baltimore Veterans Affairs Medical Center, Baltimore, MD USA; 7 R. Adams Cowley Shock Trauma Center, Department of Surgery, University of Maryland School of Medicine, Baltimore, MD USA; 8 Division of Pulmonary and Critical Care Medicine, University of Maryland School of Medicine, Baltimore, MD USA

**Keywords:** Viral infection, Viral infection

## Abstract

In light of Severe Acute Respiratory Syndrome Coronavirus 2 (SARS-CoV-2) variants potentially undermining humoral immunity, it is important to understand the fine specificity of the antiviral antibodies. We screened 20 COVID-19 patients for antibodies against 9 different SARS-CoV-2 proteins observing responses against the spike (S) proteins, the receptor-binding domain (RBD), and the nucleocapsid (N) protein which were of the IgG1 and IgG3 subtypes. Importantly, mutations which typically occur in the B.1.351 “South African” variant, significantly reduced the binding of anti-RBD antibodies. Nine of 20 patients were critically ill and were considered high-risk (HR). These patients showed significantly higher levels of transforming growth factor beta (TGF-β) and myeloid-derived suppressor cells (MDSC), and lower levels of CD4^+^ T cells expressing LAG-3 compared to standard-risk (SR) patients. HR patients evidenced significantly higher anti-S1/RBD IgG antibody levels and an increased neutralizing activity. Importantly, a large proportion of S protein-specific antibodies were glycosylation-dependent and we identified a number of immunodominant linear epitopes within the S1 and N proteins. Findings derived from this study will not only help us to identify the most relevant component of the anti-SARS-CoV-2 humoral immune response but will also enable us to design more meaningful immunomonitoring methods for anti-COVID-19 vaccines.

## Abstract (web summary)

Atanackovic and coauthors screen COVID-19 patients for antibodies against 9 different SARS-CoV-2 proteins observing responses against the spike (S) proteins, the receptor-binding domain (RBD), and the nucleocapsid (N) protein which were of the IgG1 and IgG3 subtypes. They also characterised immune responses in these patients and their findings will help to identify the most relevant component of the anti-SARS-CoV-2 humoral immune response as well as assist the design of immunomonitoring methods for anti-COVID-19 vaccines.

## Introduction

The outbreak of COVID-19 and its rapid transmission around the world has resulted in a global health emergency^1^ with the pandemic having become the greatest health challenge worldwide^2^. Clinical presentations range from asymptomatic disease to acute respiratory-distress syndrome (ARDS) and death^3,4^. Patients at an advanced age with pre-existing medical conditions typically show a more-severe disease and a worse prognosis^5–9^.

The COVID-19 infection is caused by a novel coronavirus, Severe Acute Respiratory Syndrome Coronavirus 2 (SARS-CoV-2)^10^. The SARS-CoV-2 virus contains various non-structural proteins and four major structural proteins: surface-exposed spike (S), membrane (M), envelope (E), and the internal nucleocapsid (N) proteins^10,11^. The S fusion protein consists of the S1 and S2 components and the virus enters cells, such as pneumocytes in the lung^12^, via binding of the receptor-binding domain (RBD) within the S1 protein^13^, to the angiotensin-converting enzyme-2 (ACE2) receptor^11,14^.

The infection potentially results in the formation of SARS-CoV-2-specific CD8^+^ cytotoxic T cells, which can directly target infected cells, as well as CD4^+^ T-helper cells that are able to support the formation of antigen-specific B cells and anti-SARS-CoV-2 antibody production. This adaptive immune response has the potential to control viral infection and improve patient outcomes. Our group and others have recently shown that most patients with COVID-19^15^ indeed develop spontaneous antibody-mediated immune responses against viral proteins^11,16–18^. Importantly, long-term studies suggest that sufficient levels of anti-COVID antibodies are associated with protection from future COVID-19 infections^19,20^.

Recently, three vaccines were approved for the prevention of COVID-19^21,22^ and all three have been shown to elicit antibody- and T cell-mediated antiviral immune responses conferring almost complete protection against COVID-19^23–28^. However, especially in light of the recent occurrence of variants of SARS-CoV-2 containing mutations potentially undermining antibody-mediated immunity^29–36^, it is more important than ever to understand the fine specificity of the protective humoral immune responses against SARS-CoV-2. Here, we report on our in-depth analysis of polyclonal humoral immune responses in patients with COVID-19. Findings derived from this study, including the identification of immunodominant B-cell epitopes, will not only help us to delineate the most relevant component of the antiviral humor response but will also enable us to design more meaningful methods of monitoring immune responses following anti-COVID-19 vaccination.

## Results

### COVID-19 results in IgG, IgM, and IgA antibody responses against distinct SARS-CoV-2 proteins

**Fig. 1:**
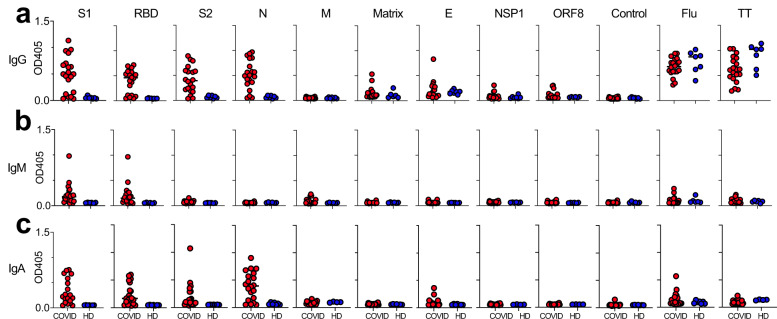
Analysis of antibody responses against full-length proteins. Analyzing plasma samples from 20 patients with COVID-19 in an ELISA we observed **a** IgG, **b** IgM (middle), and **c** IgA responses against a restricted group of four full-length recombinant proteins of the SARS-CoV-2 virus. Full-length GST (glutathione S-transferase) protein was used as a negative control and Influenza A nucleoprotein (Flu) and tetanus toxoid (TT) proteins served as positive controls. Dots indicate resulting OD values of patients (red) vs 7 healthy controls (blue). Asterisks indicate statistical significance of differences as determined by unpaired two-tailed Student’s *t* test. *p < 0.05; **p < 0.01; ***p < 0.001; ns: not significant.

As a first step, we screened 20 patients who were admitted to the University of Maryland for COVID-19 for the presence of IgG, IgM, and IgA antibodies against a total of nine different proteins of the SARS-CoV-2 virus. Plasma samples were collected at a median of 10 days (range 6–39) for the high-risk group and 7 days (range 2–15) for the standard risk group (*p* = n.s). Samples were analyzed using full-length recombinant protein in an enzyme-linked immunosorbent assay (ELISA). Importantly, no reactivity was seen with sera collected from seven healthy donors (HD) collected prior to the emergence of COVID-19 for most of the SARS-CoV-2 proteins with the exception of the matrix and E proteins where minimal background levels were observed (Fig. 1). Strong IgG responses (Fig. 1a), but no IgM (Fig. 1b) or IgA (Fig. 1c) responses, were observed in both HDs and COVID-19 patients against control antigens Influenza A nucleoprotein (Flu) nucleoprotein and tetanus toxoid (TT).

In our patients with COVID-19, no convincing antibody responses were observed against five of the nine SARS-CoV-2 proteins used during the screening part of this study (Fig. 1). However, the majority of all patients evidenced IgG (Fig. 1a) and IgA (Fig. 1c) antibody responses against the S1, RBD, S2, and N viral proteins. In addition, some of the patients also evidenced IgM (Fig. 1b) responses against the S1 and RBD proteins. Based on these findings, we decided to focus on antibody responses against the S1, RBD, S2, and N proteins of the SARS-CoV-2 virus for the remainder of this study.

**Fig. 2:**
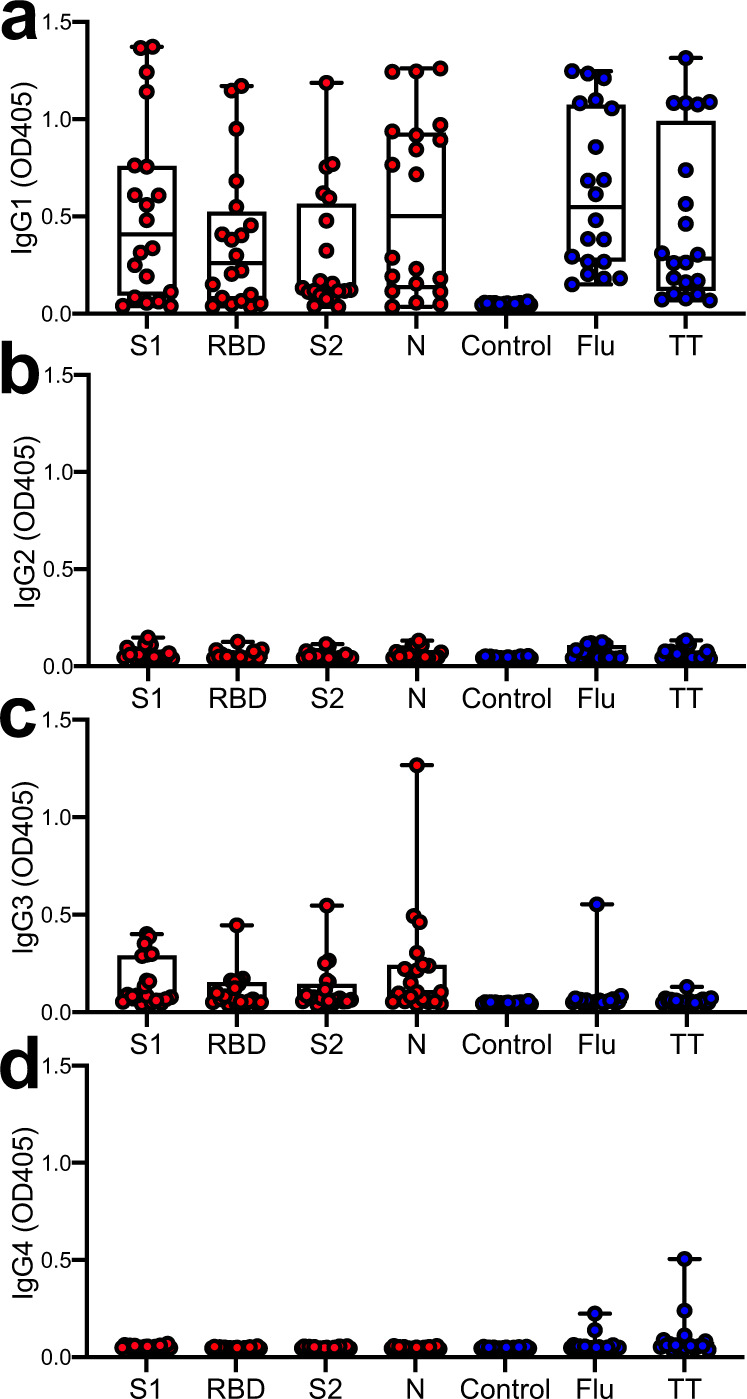
Analysis of IgG subclasses in seropositive patients. Analyzing plasma samples from 20 patients with COVID-19 for IgG responses against the four SARS-CoV virus proteins S1, RBD, S2, and N in an ELISA we found that these responses primarily consisted of the **a** IgG1 and **c** IgG3 but not the **b** IgG2 and **d** IgG4 subtypes. Full-length GST protein was used as a negative control and Flu and TT proteins served as positive controls. Boxplots extend from the 25th to 75th percentiles, the line indicates the median, and whiskers indicate the range.

In the next step, we aimed at determining the IgG subtypes elicited by the different SARS-CoV-2 proteins. We found that most of the polyclonal IgG antibodies against the 4 SARS-CoV-2 proteins detected in our patients were of the IgG1 subtype (Fig. 2a). In addition, some of the anti-S1, -RBD, -S2, and -N antibodies were of the IgG3 subtype (Fig. 2c). No IgG2 (Fig. 2b) or IgG4 (Fig. 2d) antibody responses were detected against any of the SARS-CoV-2 proteins.

### Mutations occurring in the South African variant of SARS-CoV-2 diminish binding of polyclonal antibodies from COVID-19 patients to the RBD protein

**Fig. 3:**
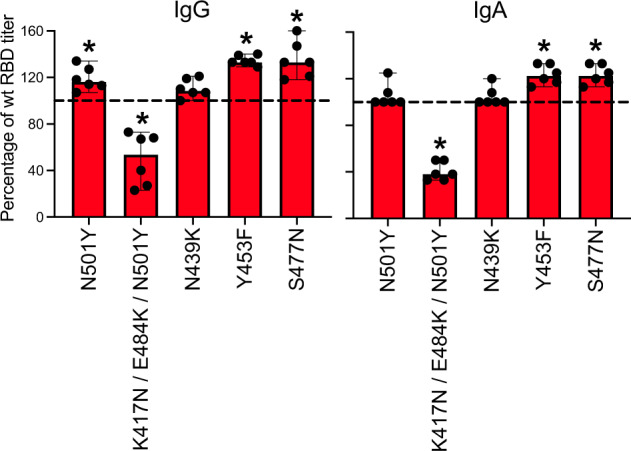
Antibody responses against SARS-CoV-2 variants. We analyzed plasma samples from six patients with known antibody response against the native RBD protein for binding of their polyclonal IgG (left) and IgA (right) antibodies to RBD proteins harboring distinct mutations typically found in different SARS-CoV variants. Binding of polyclonal sera was significantly reduced compared to the wildtype RBD (dashed line) in the case of the RBD protein containing three different mutations K417N/E484K/N501Y. Bar graphs indicate medians and whiskers indicate 95% C.I. Asterisks indicate significance levels of *p* < 0.05 when compared to responses against the native RBD protein in the same patient using a Wilcoxon test.

The 501Y.V2 variant of SARS-CoV-2, also known as the South African COVID-19 variant or B.1.351 variant, is able to attach more easily to human cells expressing the ACE2 receptor because of three mutations in the RBD: K417N, E484K, and N501Y. Two of these mutations, E484K and N501Y, are within the receptor-binding motif (RBM) of the RBD. We investigated whether these mutations, as well as other mutations occurring in different variants of SARS-CoV-2, affect the binding of the polyclonal sera from our COVID-19 patients to the RBD, potentially resulting in reduced protection from future infections with variants of the virus. Interestingly, when we analyzed the serum of 6 of our patients with relatively high antibody titers against the wildtype RBD protein, we found that isolated mutations such as N501Y, Y453F or S477N seemed to even slightly increase the binding of our patients’ polyclonal anti-RBD IgG and IgA antibodies when compared with binding to the original RBD protein (Fig. 3). In contrast the combination of K417N, E484K, and N501Y mutations, which typically occur in the 501Y.V2 South African variant, significantly reduced the binding of the polyclonal IgG and IgA antibodies to the RBD protein (Fig. 3).

### More symptomatic and inflammatory COVID-19 correlates with more pronounced anti-SARS-CoV-2 antibody responses

Out of all 20 patients, a total of nine patients had to be admitted to the intensive care unit because they were critically ill (Table 1) and were thus classified as clinically high-risk (HR). Compared with the standard risk (SR) patients, HR patients were significantly more obese and required more intensive treatment including mechanical ventilation, Extracorporeal Membrane Oxygenation (ECMO), and vasopressors. As a result of their more-severe disease, HR patients were hospitalized significantly longer (Table 1). On the day the sample for the assessment of antiviral antibodies was collected, HR patients showed higher white blood cell and neutrophil counts and a significantly increased neutrophil/lymphocyte ratio (Table 1).

**Table 1:** Patient characteristics.

	High risk (HR)	Standard risk (SR)	*P* value
**Total number**	9	11	
*Demographics*			
Age (years)	51 (26–76, IQR: 22)	66 (31–75, IQR: 29)	n.s.
Male sex	2 (22.2%)	5 (45.5%)	n.s.
Ethnicity			n.s.
African American	5 (55.6%)	8 (72.7%)	n.s.
Hispanic	3 (33.3%)	1 (9.1%)	n.s.
Caucasian	1 (11.1%)	2 (18.2%)	n.s.
*Clinical Characteristics*			
Collection (days post PCR)^*^	10 (6–39, IQR: 9.5)	7 (2–15, IQR: 3)	n.s.
Body mass index (kg/m^2^)	33.3 (28.2–45.8, IQR: 12.9)	28.3 (17.5–37.8, IQR: 11.3)	0.018
Charlson Comorbidity Index	2 (0–5)	5 (1–10)	n.s.
Mechanical Ventilation (yes/no)	9 (100%)	1 (9.1%)^**^	0.001
ECMO (yes/no)	3 (33.3%)	0	0.001
Vasopressors (yes/no)	8 (88.9%)	0	0.001
Renal dysfunction^+^ (yes/no)	3 (33.3%)	4 (36.4%)	n.s.
Renal dysfunction requiring RRT (yes/no)	1 (11.1%)	0	n.s.
Transaminitis >1.5 UL (yes/no)	7 (77.8%)	4 (36.4%)	n.s.
COVID-19-directed therapy (yes/no)	7 (77.8%)	5 (45.5%)	n.s.
Remdesivir^#^	7 (77.8%)	5 (45.5%)	
Corticosteroids^#^	5 (55.6%)	1 (9.1%)	
Convalescent plasma^#^	3 (33.3%)	1 (9.1%)	
Other***	2 (22.2%)	4 (36.4%)	
Laboratory values (day of collection)			
WBC (K/mcL)	9.9 (6.7–20.1, IQR:8.3)	6.6 (0.6–12.4, IQR:4.3)	0.007
ALC (K/mcL)	1.3 (0.4–2.8)	1.2 (0.3–1.9)	n.s.
ANC, median (K/mcL)	8.3 (3.3–14.6, IQR:5.4)	3.1 (0.03–6.7, IQR:2.2)	0.002
ANC/ALC ratio	6.3 (4.4–20.8, IQR:3.4)	2.2 (0.1–6.1, IQR:3.2)	0.001
Hemoglobin (g/dL)	9.3 (503–11.7)	8.0 (6.9–13.8)	n.s.
Platelets (K/mcL)	262 (120–648)	217 (10–319)	n.s.
CRP (mg/dL)	12.7 (1.9–28)	7.1 (1.3–20.5)	n.s.
Ferritin (ng/mL)	427.6 (131–1757.5)	449.3 (38.1–5872.3)	n.s.
d-dimer (ng/mL)	2295 (690–18800)	2070 (700–4850)	n.s.
INR	1.1 (1.1–1.3)	1.1 (1–3.4)	n.s.
PTT (seconds)	33.4 (29–70)	52 (29–196)	n.s.
*Clinical outcomes*			
Length of stay (days)	43 (7–127, IQR: 84.5)	11 (4–19, IQR:6)	0.002
Disposition home	4 (44.4%)	7 (63.6%)	n.s.
Death due to COVID-19	1 (11.1%)	0	n.s.

Numbers represent median values and ranges in brackets or absolute numbers and percentages in brackets. IQR: Interquartile range, for select variables. Numbers were compared between groups using a Mann–Whitney *U* or Chi-Square test (n.s.=not significant).

*ECMO* extracorporeal membrane oxygenation, *RRT* renal replacement therapy, *WBC* white blood cell count, *ALC* absolute lymphocyte count, *ANC* absolute neutrophil count, *CRP* C-reactive protein.

*Defined as collection day from first positive SARS-CoV-2 PCR.

**Mechanical ventilation for a surgical procedure, not due to COVID-19.

^+^Renal dysfunction defined as creatinine >0.3 mg/dl from baseline or creatinine >1.5 mg/dl.

***Six patients with “other” as therapy designation included four patients receiving Tocilizumab on a clinical trial and two patients enrolled on a placebo-controlled trial (Baricitinb vs placebo, BLD-2660 vs placebo).

^#^10 out of 12 patients who received remdesivir were already on remdesivir at the time of sample collection for this study. Sample collection and analysis occurred prior to steroid administration (*n* = 6). All four patients who received convalescent plasma as a therapy, received it after the enrollment on this sample collection (*n* = 4).

**Fig. 4:**
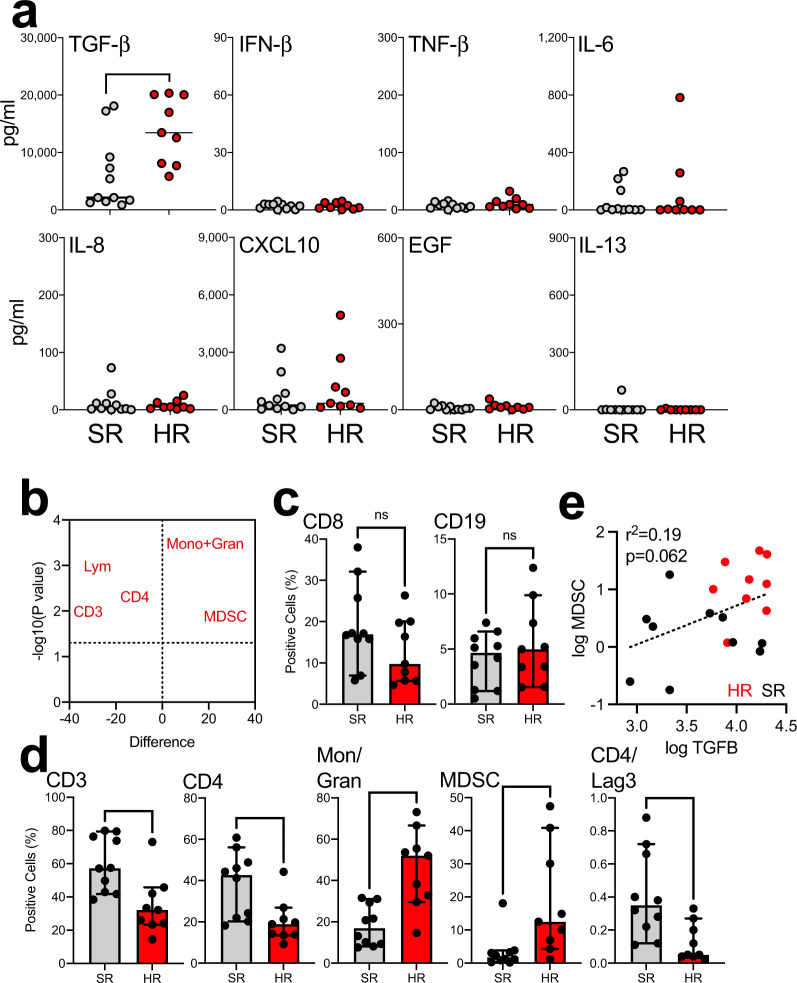
Analysis of cytokine patterns and T cell phenotypes in COVID-19 patients. **a** We analyzed cytokine/chemokine levels in the peripheral blood of nine COVID patients who were critically ill (HR = high risk; red dots) and compared them to levels in 11 non-critically ill patients (SR = standard risk; gray dots). **b** Different lymphocyte subpopulations were quantified in the peripheral blood of our COVID patients using multicolor flow cytometry and subsets discriminating between HR and SR patients were identified using a volcano plot analysis. **c** No significant differences were detected with regard to two different subsets of interest: CD19+ B cells (data indicate percentages of all lymphoctes) and CD8+ T cells (data indicate percentages of all CD3^+^ lymphoctes). **d** Patients with HR disease showed lower proportions of total CD3+ T cells (data indicate percentages of all lymphoctes), CD4+ T cells (data indicate percentages of all CD3^+^ lymphoctes), and Lag-3-expressing CD4+ T cells (data indicate percentages of all CD3^+^CD4^+^ lymphoctes) but higher percentages of MDSC (data indicate percentages of all CD16^−^HLA-DR^−^ monocytes/granulocytes). Numbers represent percentages of cells within the morphologic lymphocyte or monocyte gates, respectively. Groups were compared using a Mann–Whitney *U* test (*p < 0.05, **p < 0.01, ***p < 0.001). **e** Proportions of MDSC plotted against TGF-β concentrations; r2 and *p* value were determined by linear regression.

Trying to further define the inflammatory status in the HR vs. SR patients, we first performed an analysis of levels of different cytokines/chemokines in the blood of our COVID-19 patients. Out of all eight cytokines/chemokines analyzed, only transforming growth factor-beta (TGF-β), which typically shows only very low levels in healthy individuals^37^, was present at significantly higher levels in our HR patients compared to SR patients (Fig. 4a). Next, we performed multicolor flow cytometry on our patient’s PBMCs staining for a total of 29 different markers (Supplementary Table 1). Comparing individual PBMC subsets (Fig. 4b), we were able to identify significant differences between the SR and HR groups. There were no significant differences between both groups with regard to percentages of CD8^+^ T cells and CD19^+^ B cells (Fig. 4c). However, morphological gating of the cells revealed that HR patients had significantly lower proportions of total CD3^+^ T cells and CD4^+^ T cells. In addition, the same patients showed higher percentages of myeloid-derived suppressor cells (MDSC) and lower levels of CD4^+^ T cells expressing the co-inhibitory marker LAG-3 (Fig. 4d). Importantly, the immunosuppressive function of MDSC is partially mediated by TGF-β and when we plotted levels of this cytokine, which we found to be significantly upregulated in HR patients (*p* = 0.0184), against percentages of MDSC, there was a clearly identifiable population of HR patients that showed both high proportions of MDSC and high concentrations of TGF-β (Fig. 4e).

**Fig. 5:**
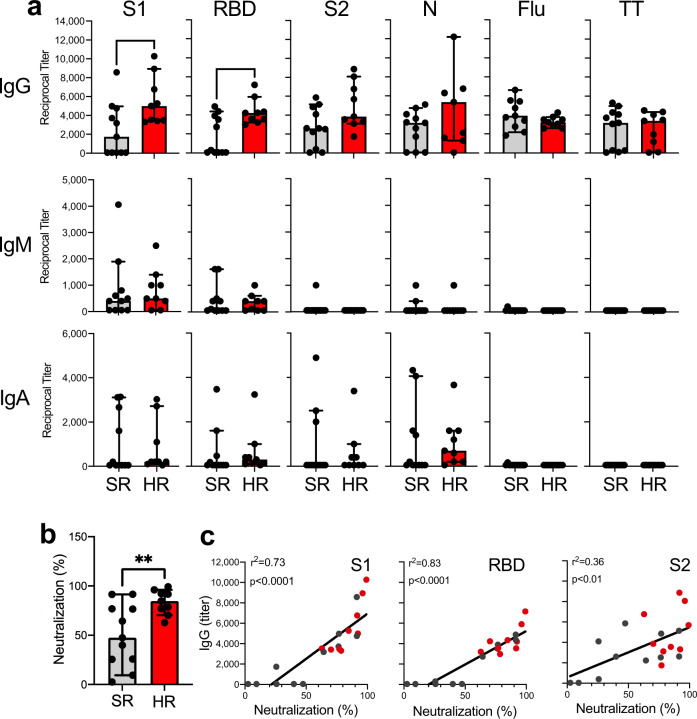
Quantification of antibody titers against SARS-CoV proteins. **a** We performed a comparative analysis IgG, IgM, and IgA antibody titers against the SARS-CoV virus proteins S1, RBD, S2, and N in nine high-risk (HR, red bars) vs. 11 standard-risk (SR, gray bars) patients. Recombinant Flu and TT proteins served as controls. Bar graphs indicate medians and whiskers indicate ranges. Asterisks indicate significance differences between groups (*p < 0.05, **p < 0.01). **b** Comparative analysis of viral neutralization in patients with high-risk vs. standard-risk COVID-19. **c** Correlation of viral neutralization with IgG antibody titers against S1, RBD, and S2 performing linear regression.

Given the differences in the number and distribution of peripheral leukocytes, particularly of helper T cells and potentially immunosuppressive cell types, we next asked the question of whether these findings would have consequences for the humoral anti-SARS-CoV-2 immunity. Comparing titers of antiviral IgG, IgM, and IgA antibodies between SR and HR patients, we indeed found that HR patients evidenced significantly higher levels of IgG antibodies against proteins S1 and RBD compared to SR patients (Fig. 5a). Importantly, HR patients showed levels of anti-Flu antibodies, anti-TT antibodies (Fig. 5a) and total IgA levels (Supplementary Figure 2) similar to SR patients (Fig. 5a) and levels of total IgG were even lower in the HR group compared to SR patients (Supplementary Figure 2).

Next, we asked the question whether the higher absolute levels of antibodies binding to S1 and RBD in HR patients would have consequences for viral neutralization in the same patient group. To measure neutralizing activity, we used a surrogate assay that detects circulating antibodies against SARS-CoV-2 inhibiting the interaction between the RBD with the ACE2 cell surface receptor. When we compared viral neutralization activity between patient groups, we found that the plasma from HR patients indeed show a significantly higher neutralizing activity based on the binding of their antibodies to the RBD protein (Fig. 5b). Accordingly, in the complete patient population, there was a very strong correlation between titers of IgG antibodies against the S1 and RBD proteins with the neutralizing activity of the patients’ plasma (Fig. 5c). In contrast, titers against S2 were only weakly correlated with neutralizing activity.

### Anti-SARS-CoV-2 antibodies target glycosylated residues of the S1 and S2 proteins and a broad range of linear epitopes in the S and N proteins

**Fig. 6:**
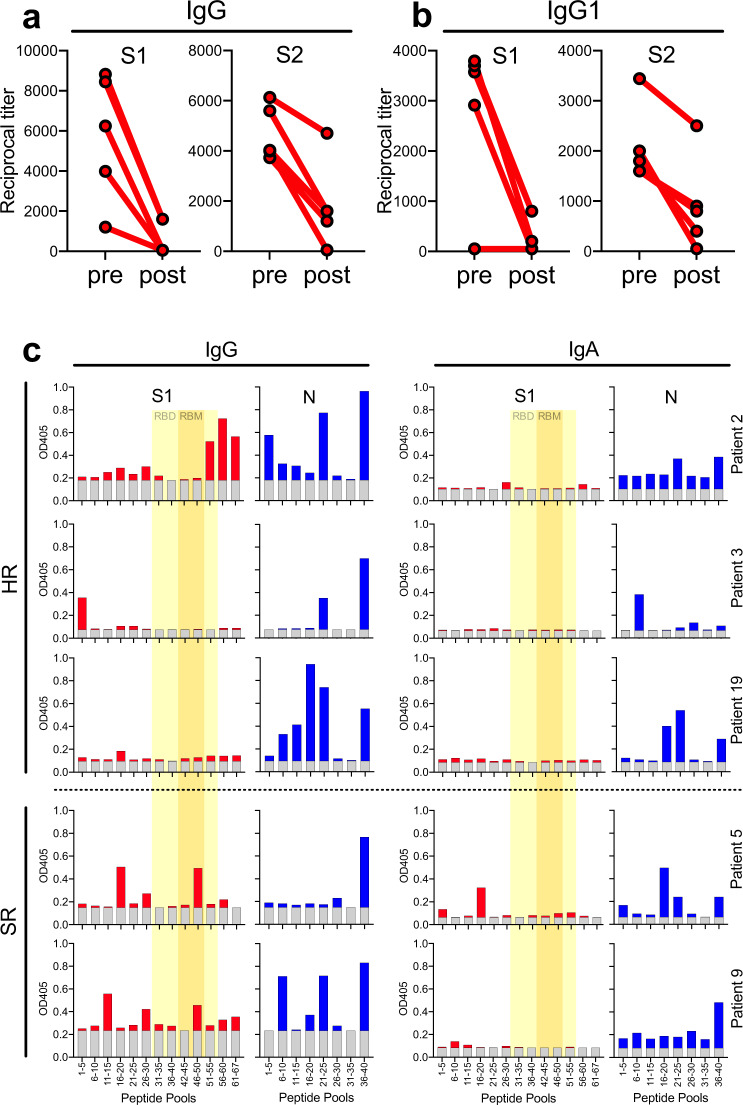
Relevance of glycosylated and linear SARS-CoV epitopes. Plasma samples from five patients with known anti-SARS-CoV reactivity were analyzed for **a** IgG and **b** IgG1 titers against deglycosylated vs. native S1 and S2 proteins. **c** We used peptide pools of five 20mer peptides each overlapping by 10aa to determine IgG and IgA reactivity against distinct regions within the S1 (red bars) and N (blue bars) proteins. Gray bars indicate background levels. RBD and RBM regions within the S1 protein are highlighted in yellow and orange, respectively. Asterisks indicate statistical significance of differences as determined by paired two-tailed Student’s *t* test. *p < 0.05; **p < 0.01.

In order to determine whether glycosylation affects antibody binding to heavily glycosylated SARS-CoV-2 S1 and S2 proteins, we deglycosylated both proteins and determined the change in their molecular weights by sodium dodecyl sulphate–polyacrylamide gel electrophoresis (SDS-PAGE). As expected, deglycosylation resulted in substantially reduced molecular weight of both proteins (Supplementary Figure 3). Next, we analyzed sera from five of our COVID-19 patients who had shown relatively high antibody titers against the wildtype S1 and S2 proteins, for reactivity with both the native and the deglycosylated S1 and S2 proteins. We found that deglycosylation resulted in substantially reduced binding of antiviral IgG (Fig. 6a) and IgG1 (Fig. 6b) antibodies to both proteins, demonstrating that a relatively large proportion of S protein-specific antibodies in individuals with anti-COVID, while not necessarily glycosylation-specific, appear glycosylation-dependent.

Next, we tried to identify individual target epitopes of S1 and N-specific polyclonal IgG and IgA antibodies in patients with SR and HR COVID-19. When we used individual pools of peptides consisting of five 20mer peptides overlapping by 10 amino acids (aa) in an ELISA, we were able to identify regions in both proteins that were preferentially targeted by the antiviral antibodies. For anti-S1 antibodies, there were regions within the RBM corresponding to peptide pool 46–50 (aa 451–510) and C-terminal of the RBD that were preferentially targeted (Fig. 6c). Antibodies specific for N protein preferentially targeted (Fig. 6C) peptide pools 16–20 (aa 151–210), 21–25 (aa 201–260), and 36–40 (aa 351–410). Overall, patients with HR COVID-19 appeared to show a broader anti-N immunity than patients with less-severe SR disease.

**Fig. 7:**
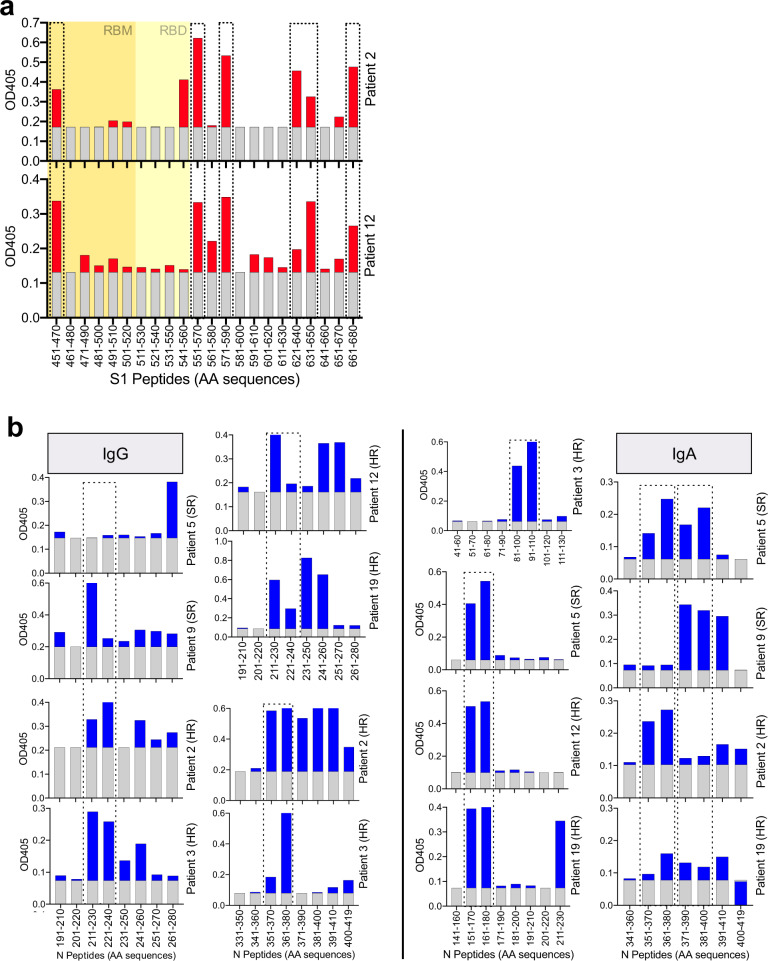
Detailed characterization of the B cell epitope landscape in COVID-19 patients. **a** Plasma samples from two patients with known SARS-CoV reactivity were analyzed for IgG responses against individual 20mer peptides of the S1 protein covering the RBD (highlighted in yellow), the RBM within the RBD (highlighted in orange), and the region C-terminally adjacent to the RBD. **b** Plasma samples from six patients with known SARS-CoV reactivity were analyzed for IgG and IgA responses against individual 20mer peptides of the N protein. Values represent ODs in an ELISA assay and shared epitopes are marked using dotted lines.

Based on the findings above, we decided to focus on the immunodominant regions within both proteins to identify individual target epitopes. Using individual 20mer peptides covering the respective regions within both proteins, we were able to describe a prominent N-terminal epitope within the RBD of the S protein at aa positions 451–470, another epitope at the C-terminal end of the RBD (aa 541–560), as well as several shared epitopes adjacent to the C-terminal end of the RBD (Fig. 7a). Interestingly, the same shared regions at the C-terminal end of the RBD were recently described as immunodominant by Shrock et al.^38^. IgG and IgA antibodies targeting the N-protein, especially when it comes to patients with HR disease, were much more broadly distributed (Fig. 7b). However, there were still some prominent epitopes shared by the majority of patients such as regions corresponding to aa 211–240 and aa 351–380 for IgG antibodies as well as aa 151–180, aa 351–380, and aa 371–400 for IgA antibodies (Fig. 7b), the latter of which was also described by Shrock et al.^38^.

## Discussion

Research has focused primarily on the spike protein of the SARS-CoV-2 virus as a potential target for antiviral immune responses as the RBD within that protein is responsible for viral entry and, therefore, the spread of the infection. Not surprisingly, studies of spontaneous and even vaccine-induced immune responses have shown that the RBD is indeed the target of most neutralizing antibodies against SARS-CoV-2^39–43^. We consider it of major importance, however, to identify additional targets for immunotherapies outside of the RBD within the S protein, especially as recent variants of SARS-CoV-2 have been shown to potentially undermine antiviral immune responses^29–36^ and additional variants are likely to occur in the future.

When we screened 20 patients with COVID-19 for antibody responses against a total of nine viral proteins, we found that only the S proteins, RBD, and the N protein had elicited spontaneous IgG, IgM, and IgG responses probably reflecting the superior immunogenicity of these proteins. The polyclonal IgG antibodies consisted primarily of the IgG1 and IgG3 subtypes. Importantly, in addition to their potential inhibitory effect on viral entry, these antibody subtypes are highly capable of initiating binding of IgG-coated viral proteins to different Fc-gamma receptors resulting not only in the induction of antibody-dependent cellular cytotoxicity but also uptake into antigen-presenting cells with the subsequent induction of antiviral T-cell responses against the given SARS-CoV-2 protein^44^.

When we analyzed the effect of certain mutations in the RBD region of the S1 protein, individually and combined, on the binding of our sera from patients with COVID-19, we found that only the introduction of a combination of the three mutations N501Y, K417N, and E484K into the RBD protein resulted in significantly reduced IgG and IgA antibody titers. Our findings are in line with previous observations that the “South African” B.1.351 variant of SARS-CoV-2, which carries the same RBD mutations, is associated with reduced viral neutralization using convalescent sera and samples from vaccinated subjects, respectively^45,46^. Our data also support the attribution of reduced binding of patient sera to the B.1.351 variant to the E484K mutation^45^ because the N501Y alone, which is located within the RBM just like E484K, did not result in reduced binding. Accordingly, prior studies have not observed an effect of just the N501Y mutation, which is the only one of these three mutations present in the “UK variant” B.1.1.7, on viral neutralization^45,46^ and introduction of the E484K mutation into a B.1.1.7 background led to a more substantial loss of neutralizing activity by vaccine-elicited antibodies^32^.

Comparing humoral immunity to SARS-CoV-2 in a group of COVID-19 patients with high-risk to a group with the standard-risk disease, we first aimed at defining each group in-depth on a clinical and immunological level, respectively. From a clinical perspective, HR patients showed a number of characteristics that have previously been identified as central risk factors for a more-severe and complicated course of COVID-19 such as obesity^4,47^. From a global immunological perspective, HR patients showed higher neutrophil counts and a significantly increased neutrophil/lymphocyte ratio, both of which have previously been described as risk factors in COVID-19^48^. At the same time, we observed higher percentages of MDSC and higher concentration of TGF-β in HR compared with SR patients, a phenomenon that has been observed before^49^ and probably represents a counterregulatory mechanism in this highly inflammatory immune environment. Importantly, the immunosuppressive function of MDSC is partially mediated by TGF-β^50^ and the expansion of MDSC in HR patients can lead to an undesired inhibition of a SARS-CoV-2-specific T-cell response, resulting in worse outcomes in these patients^51^. Furthermore, TGF-β itself has been suggested to be involved in the promotion of COVID-19-associated pulmonary disease^52^ and, accordingly, both MDSC and TGF-β should be further investigated as pathogenic/prognostic factors and potential therapeutic targets in COVID-19.

Comparing antiviral immunity between SR and HR patients we found that critically ill patients evidenced significantly higher levels of IgG antibodies against proteins S1/RBD and these antibody titers correlated positively with the neutralizing activity of the patients’ plasma. These findings confirm previous observations indicating that Interestingly, the magnitude of antibody responses to SARS-CoV-2 seem to correlate directly with symptom severity^53–55^ and, accordingly, hospitalization for COVID-19 and/or the presence of critical disease can predict high antibody levels^56–58^. Whether these strong antibody responses in patients with high-risk COVID-19 and a prolonged course of the disease eventually result in superior protection from re-infection or whether they are even detrimental remains to be seen. In this context, it is noteworthy that patients with severe COVID-19 have been shown to produce antibodies that functionally block the production of major cellular immune components expressing interferon-stimulated genes, thereby potentially dampening cellular antiviral immune responses^59^.

The spike proteins of the SARS-CoV-2 virus, which are heavily glycosylated, have been in the spotlight of attention as targets for anti-COVID-19 immunotherapies. Aiming at defining the relevance of glycosylation for anti-SARS-CoV-2 antibody responses, we are showing that a large proportion of the total S protein-specific antibodies in our patients with anti-COVID immunity are glycosylation-dependent. Importantly, this finding may be the result of changed S protein conformation upon deglycosylation and does not necessarily reflect the presence of glycosylation-specific antibodies. These findings do not only have important implications for the design of immunotherapies targeting SARS-CoV-2 but also for the way immunomonitoring should be performed, e.g., for anti-COVID-19 vaccines.

In addition to the glycosylated residues targeted by polyclonal sera from patients with COVID-19, we also demonstrate here the reactivity of a set of patient-derived polyclonal sera to different linear epitopes within the S1 and N proteins. In addition to showing reactivity of the majority of antibodies with glycosylated S1 protein, we also observed frequent recognition of several linear non-glycosylated peptides within the RBM, RBD, NTD, and C-terminal of the RBD within the S1 protein. It has been demonstrated previously that monoclonal antibodies isolated from the B cells of patients infected with SARS-CoV-2 preferentially target the RBD region within S1, that the vast majority of those antibodies show efficient virus neutralization^42,43,60–64^, and that this neutralizing RBD-specific response is highly convergent indicating selective pressure enriching for a limited set of potent neutralizing antibodies^62^. However, it has also been shown that the neutralizing antibody response is not restricted to only the RBD^65^. Generally, S1-specific antibodies can be grouped into two major categories: (1) antibodies recognizing epitopes within the RBM, competing with ACE2 binding, and preventing virus attachment^42,43,61–64^, and (2) antibodies recognizing epitopes outside of the RBM and not competing with ACE2 binding^62,66^. Although the mechanism of neutralization by antibodies targeting regions outside of the RBM remains an active area of research, it has been suggested that binding of such antibodies may result in the locking of S1 in an open conformation^42^ or the aggregation of virions resulting in efficient virus neutralization^67^.

We would like to stress, however, that viral neutralization through antibody-mediated inhibition of viral entry is not the only relevant immune defense mechanism, in particular in patients with an ongoing severe course of the infection and a comparably high viral load such as our patients who were critically ill. In these patients, T-cell-mediated control and eventually eradication of the disease is probably even more important and we consider it possible, if not likely, that many of the polyclonal antibody responses we have described here, in particular the very broad antibody responses against different epitopes of the N protein, may support polyclonal T-cell responses against the same antigen. Future studies are needed to elucidate in detail the interplay between B-cell and T-cell responses with a focus on patients with severe COVID-19.

## Methods

### Patients and samples

We collected 40 ml of heparinized blood from 20 consecutive COVID-19 patients (Table 1) who were admitted to the University of Maryland Medical Center between June and August of 2020. Informed consent was obtained and blood samples were collected under IRB HP-00091425. Patients were enrolled in two cohorts, the high-risk group, and the standard-risk group. The high-risk group had a critical illness (requiring ventilatory support) and the standard risk group had mild, moderate, and severe illness as per National Institutes of Health COVID-19 illness severity classification^68^. Plasma was generated from peripheral blood samples after centrifugation at 400 × *g* for 10 min and frozen immediately at −80°C. Peripheral blood mononuclear cells (PBMCs) were isolated using a lymphocyte separation density gradient and immediately frozen in liquid nitrogen. Plasma samples collected from seven anonymous HDs before the COVID-19 epidemic started were used as controls.

### Proteins and peptides

We used a total of 16 full-length recombinant SARS-CoV-2 proteins and seven control proteins for our assays (Supplementary Table 1). For protein deglycosylation we used Protein Deglycosylation Mix II (New England Biolabs, Ipswich, MA) according to the manufacturer’s instructions. Glycosylated and deglycosylated proteins were subjected to SDS-PAGE and stained with GelCode Blue Safe Protein Stain (Thermo Fisher Scientific, Halethorpe, MD) to confirm efficient deglycosylation. For our peptide ELISA, we used peptide libraries consisting of biotinylated 20mer peptides (Peptides&Elephants, Hennigsdorf, Germany) overlapping by 10 aa covering the complete sequence of the respective protein. Peptides contained an N-terminal TTDS spacer followed by an SGSG linker.

### Plasma cytokine/chemokine analysis

Plasma aliquots were thawed and centrifuged at 3000 × *g* for 20 min and then at 10,000 × *g* for 30 min at 4 °C to eliminate potential cell debris and contaminants. Supernatants were used for the analysis of a panel of eight cytokines with the Milliplex® Multiplex Assay (MilliporeSigma, Burlington, MA). In brief, a 96-well plate was incubated with 200 µL of Assay Buffer for 10 min. The buffer was decanted, Assay Buffer was added to each well containing 25 µL of the sample. Then 25 µL of a mixture containing anti-cytokine antibody-conjugated beads (1:50 dilution) were added and incubated at 4 °C overnight. The plate was washed three times with Wash Buffer and incubated with 25 µL of biotin-labeled detection antibody. Later, 25 µL of streptavidin-Phycoerythrin at 1:15 dilution was added to each well and incubated for 30 min. The plate was washed and 150 µl of Sheath Fluid was added per well. The plate was read in a Luminex MagPix reader and concentrations were calculated using Luminex’s Exponent software.

### Flow cytometry

PBMC were resuspended in DPBS, stained with Zombie NIR for 15 min at room temperature. The cells were washed, blocked, and stained using a 28-antibody cocktail (Supplementary Table 2). Cells were washed, fixed and samples were then acquired on a 4-laser Cytek Aurora spectral cytometer (Cytek, Fremont, CA). Single color controls were stained on UltraComp eBeads™ Compensation Beads (ThermoFisher Scientific, Cat# 01-2222-42), fixed, and used for spectral unmixing. Events were acquired using the gating strategy described in Supplementary Figure 1. Debris was removed using the SSC-B/SSC plot and autofluorescence was removed by selecting it as a fluorescence tag during unmixing. The unmixed data were analyzed in FCS Express 7 (De Novo Software, Pasadena, CA). A clean-up gate on HLA-DR/CCR7 plot was used to gate out very high fluorescence events due to antibody aggregation.

### Enzyme-linked immunosorbent assay

Serum antibody responses were determined by ELISA as previously described^69^. High-binding ELISA plates (Thermo, cat # 44-2404-21) were coated with 5 µg/mL of the respective proteins in PBS (Gibco, cat # 10010-023) overnight at 4 °C. The next day plates were washed twice with PBS and twice with 0.1% PBS-T (VWR cat # M147-1L). Plates were then blocked with 5% non-fat dry milk (Santa Cruz, cat # sc2325) in PBS (MPBS) for 1 h at room temperature (RT) then washed again as described above. Serum was diluted 1:40 for screening assays and 1:100/1:400/1:1600/1:6400 for titrations in MPBS. Diluted sera were added to plates and incubated for 3H at RT. Plates were washed as described above before incubation with secondary antibodies against pan-human IgG (Southern Biotech, cat # 2040-04), IgG1 (Southern Biotech, cat # 9052-04), IgG2 (Southern Biotech, cat # 9060-04), IgG3 (Southern Biotech, cat # 9210-04), IgG4 (Southern Biotech, cat # 9200-04), IgA (Southern Biotech, cat # 2050-04), and IgM (Jackson ImmunoResearch, cat # 109-055-129). Secondary antibodies were diluted according to the manufacturers’ instructions and plates incubated for 1H at RT. Plates were then washed as described above, PNPP tablets (Southern Biotech, cat # 0201-01) dissolved in diethanolamine (Thermo, cat # 34064) and PNPP substrate solution added to each well for 10 min in the dark. In all, 15 μL of 3 N NaOH (VWR, cat # BDH7472-1) stop solution was added to each well, and absorbance was read at 405 nm with a reference wavelength of 620 nm. For peptide ELISAs, plates were first coated with 5ug/ml neutravidin (Thermo) overnight at 4 C and then blocked with 2% bovine serum albumin (BSA) in PBS for 1 h at room temperature. Plates were then incubated for 1 h at room temperature with either 1 μg/mL of the individual peptides or 5 μg/mL equimolar peptide pools in PBS as indicated. Plates were washed and then developed with serum at a dilution of 1:40 and with secondary reagents as described above using 2% BSA instead of MPBS. For the calculation of titers, regression analyses were performed for the linear segment of serum titration curves for positive samples and pooled sera of five HDs. For titers against TT and Flu we used recombinant GST protein as a negative control for the calculation of titers. Titers were defined as the dilution at the intersection of both regression lines.

### SARS-CoV-2 neutralization assay

Neutralizing activity of patient sera was assessed using the cPass Neutralization Antibody Detection Kit which detects circulating neutralizing antibodies against SARS-CoV-2 that block the interaction between the receptor-binding domain (RBD) of the viral spike glycoprotein with the ACE2 cell surface receptor. In brief, samples and controls were diluted with sample dilution buffer and pre-incubated with the horseradish peroxidase (HRP) conjugated recombinant SARS-CoV-2 RBD fragment (HRP-RBD) to allow the binding of the circulating neutralization antibodies to HRP-RBD. The mixture was then added to the capture plate, which was pre-coated with the hACE2 protein. The unbound HRP-RBD, as well as any HRP-RBD bound to non-neutralizing antibody, was captured on the plate, whereas the circulating neutralization antibodies HRP-RBD complexes remained in the supernatant and were removed during washing. Following a wash cycle, TMB substrate solution was added followed by the Stop Solution. The absorbance of the final solution was read at 450 nm in a microtiter plate reader (Tecan, Morrisville, NC). The degree of inhibition of RBD–AC2 interactions was calculated in relation to the positive control leading to complete inhibition.

### Statistical analyses

Statistical analyses were performed using GraphPad Prism 9 software (GraphPad Software, San Diego, CA). A two-tailed unpaired Student’s *t* test was used to determine the statistical significance of differences in antigen binding between sera from HDs and patients, as well as patients with standard-risk and high risk. Differences in cell percentages and cytokine levels were determined by Mann–Whitney U test. Differences in binding to mutant S protein variants were analyzed by Wilcoxon test. To determine the statistical significance of differences in binding of glycosylated and deglycosylated S proteins by patient sera, two-tailed paired Student’s *t* test was used. Linear regression was used to determine the statistical significance of the association between IgG titers and neutralization as well as MDSC percentage and TGFB levels. Results were considered significant when *p* < 0.05.

### Reporting summary

Further information on research design is available in the Nature Research Reporting Summary linked to this article.

## Supplementary information

Supplementary Information

Description of Additional Supplementary Files

Supplementary Data 1

Reporting Summary

## Data Availability

The data that support the findings of this study are available from the corresponding author upon reasonable request and all the data used for our analyses and figures are available as part of the Supplementary Materials as a data set named Supplementary Data 1.
