# Supplementary material for: Deep dissection of the antiviral immune profile of patients with COVID-19
Source: Commun Biol. 2021 Dec 16;4:1389. doi: 10.1038/s42003-021-02852-1 (PMC8677724; doi:10.1038/s42003-021-02852-1)
Supplement: Supplementary file 1 — Supplementary Information [file 42003_2021_2852_MOESM1_ESM.pdf]

Supplementary Figure 1

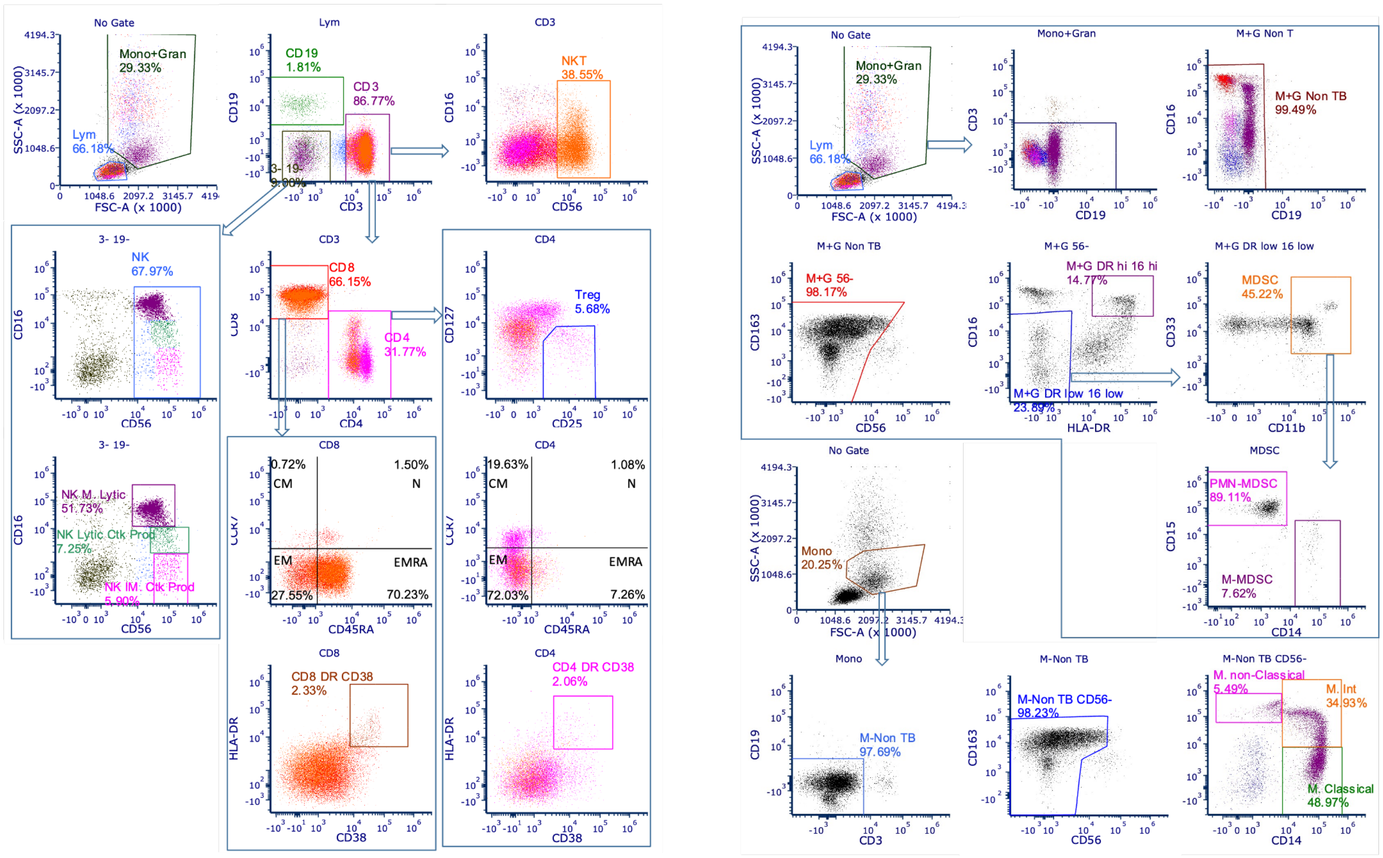

<u>Supplementary Figure 1:</u> Gating strategy for analyzing COVID-19 samples using flow cytometry.

Supplementary Figure 2

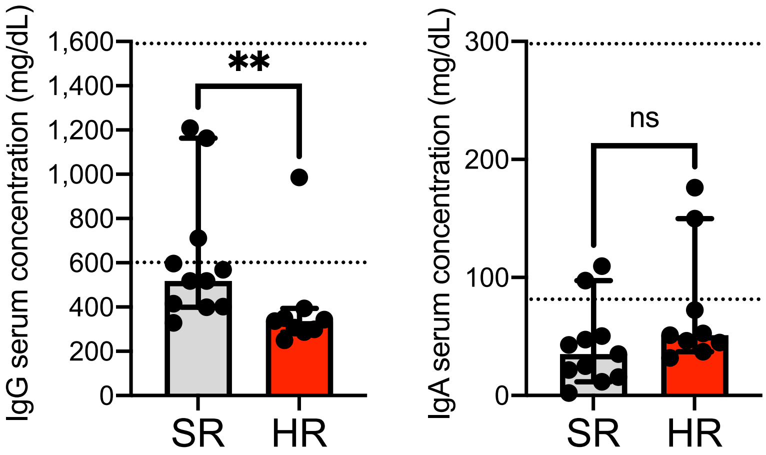

<u>Supplementary Figure 2:</u> Absolute IgG and IgA levels in patients with COVID-19
Absolute concentrations of IgG and IgG were determined in the peripheral blood of patients with standard risk (SR) and high risk (HR) disease using an ELISA. Asterisks indicate significant differences between groups using a Mann-Whitney U test (**p<0.01).

Supplementary Figure 3

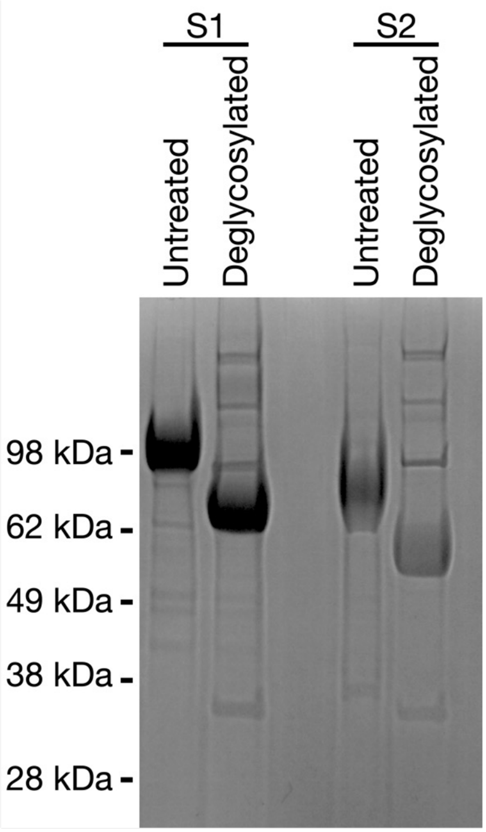

<u>Supplementary Figure 3:</u> **Deglycosylation of SARS-CoV-2 S1 and S2 proteins**
Both SARS-CoV-2 proteins were deglycosylated and their molecular weight was determined by SDS-PAGE.

SUPPLEMENTARY TABLES

Supplementary Table 1: Proteins used in ELISA

Protein	Abbreviation	Vendor	Expression System
Epstein-Barr Virus Glycoprotein gp350	EBV	Sino Biological	Baculovirus Insect Cells
SARS-CoV-2 Envelope	E	Acro Biosystems	E. coli
Influenza A H1N1 Nucleoprotein	Flu	Sino Biological	Baculovirus-Insect Cells
Human Cytomegalovirus Glycoprotein B	CMV	Sino Biological	HEK293
SARS-CoV-2 Matrix	Matrix	BioVision	E. coli
SARS-CoV-2 Membrane	M	BioVision	E. coli
SARS-CoV-2 Nonstructural Protein 1	NSP1	Acro Biosystems	E. coli
SARS-CoV-2 Nucleoprotein	N	BioVision	E. coli
SARS-CoV-2 Nucleoprotein	N	Acro Biosystems	HEK293
SARS-CoV-2 ORF8	ORF8	Invitrogen	E. coli
SARS-CoV-2 Receptor Binding Domain	RBD	Invitrogen	HEK293
SARS-CoV-2 S1	S1	Acro Biosystems	HEK293
SARS-CoV-2 S2	S2	Acro Biosystems	HEK293
Tetanus Toxoid	TT	Boehringer	
Herpes Simplex Virus 1 gD (266-394)	HSV	abcam	E. coli
Herpes Simplex Virus 1 gG	HSV	abcam	E. coli
SARS-CoV-2 RBD (N501Y)	RBD	Acro Biosystems	HEK293
SARS-CoV-2 RBD (K417n, E484k, N501Y)	RBD	Acro Biosystems	HEK293
SARS-CoV-2 RBD (N439K)	RBD	Acro Biosystems	HEK293
SARS-CoV-2 RBD (Y453F)	RBD	Acro Biosystems	HEK293
SARS-CoV-2 RBD (S477N)	RBD	Acro Biosystems	HEK293
SARS-CoV-2 Receptor Binding Domain	RBD	Acro Biosystems	HEK293
Glutathione S-transferase	GST	House Made	Expi293

Supplementary Table 2: Monoclonal antibodies used for flow cytometry

Marker	Fluorochrome	Isotype	Clone	Company
**CD62L**	BUV395	IgG2a κ	SK11	BD Biosciences
**CD8**	BUV496	IgG1 κ	RPA-T8	BD Biosciences
**CD56**	BUV563	IgG2b κ	NCAM16.2	BD Biosciences
**CD11b**	BUV661	IgG2b κ	M1/70	BD Biosciences
**CD4**	BUV805	IgG1 κ	SK3	BD Biosciences
**CD127**	BV 421	IgG1	A019D5	BioLegend
**CD19**	Pacific Blue	IgG1	SJ25C1	BioLegend
**CD14**	BV480	IgG2b κ	MφP9	BD Biosciences
**CD40L**	BV 510	IgG1	24-31	BioLegend
**CD16**	BV 570	IgG1	3G8	BioLegend
**TIM-3**	BV 605	IgG1 κ	F382E2	BioLegend
**CCR7**	BV 650	IgG2a	G043H7	BioLegend
**CD163**	BV 711	IgG1	GHI/61	BioLegend
**CD45RO**	BV 750	IgG2a	UCHL1	BioLegend
**CD15**	BV 785	IgG1	W6D3	BioLegend
**HLA-DR**	FITC	IgG1	L243	BioLegend
**CD3**	Spark 550	IgG1	SK7	BioLegend
**PD-1**	PE	IgG1	EH12.2H7	BioLegend
**CD94**	PE-Dazzle 594	IgG1	DX22	BioLegend
**CD25**	PE-Cy5	IgG1	BC96	BioLegend
**CD45RA**	PerCP	IgG2b	HI100	BioLegend
**CD147**	PerCP-Cy5.5	IgG1	HIM6	BioLegend
**Lag-3**	PE-Cy7	IgG1	11C3C65	BioLegend
**CD137**	APC	IgG1	4B4-1	BioLegend
**CD33**	Alexa 647	IgG1	P67.6	BioLegend
**CD95**	Alexa 700	IgG1	DX2	BioLegend
**CD27**	APC/Fire 750	IgG1	O323	BioLegend
**CD38**	APC/Fire 810	IgG2	HIT2	BioLegend
**Zombie NIR**				BioLegend
