# Supplementary material for: Deep dissection of the antiviral immune profile of patients with COVID-19
Source: Commun Biol. 2021 Dec 16;4:1389. doi: 10.1038/s42003-021-02852-1 (PMC8677724; doi:10.1038/s42003-021-02852-1)
Supplement: Supplementary file 2 — Description of Additional Supplementary Files [file 42003_2021_2852_MOESM2_ESM.pdf]

Description of Additional Supplementary Files

File name: Supplementary Data 1.
Description: Complete set of all data used for analysis and figures.
